# Open-lung ventilation versus no ventilation during cardiopulmonary bypass in an innovative animal model of heart transplantation

**DOI:** 10.1186/s40635-024-00669-w

**Published:** 2024-11-27

**Authors:** Varun Karnik, Sebastiano Maria Colombo, Leah Rickards, Silver Heinsar, Louise E. See Hoe, Karin Wildi, Margaret R. Passmore, Mahe Bouquet, Kei Sato, Carmen Ainola, Nicole Bartnikowski, Emily S. Wilson, Kieran Hyslop, Kris Skeggs, Nchafatso G. Obonyo, Charles McDonald, Samantha Livingstone, Gabriella Abbate, Andrew Haymet, Jae-Seung Jung, Noriko Sato, Lynnette James, Benjamin Lloyd, Nicole White, Chiara Palmieri, Mark Buckland, Jacky Y. Suen, David C. McGiffin, John F. Fraser, Gianluigi Li Bassi

**Affiliations:** 1 Critical Care Research Group, The Prince Charles Hospital, Level 3, Clinical Sciences Building, Chermside Qld 4032, Brisbane, QLD Australia; 2 Griffith University School of Medicine, Gold Coast, Australia; 3 Faculty of Medicine, University of Queensland, Brisbane, QLD Australia; 4 Department of Anesthesia, Critical Care and Emergency, Fondazione IRCCS Ca’ Granda, Ospedale Maggiore Policlinico, Milan, Italy; 5 Department of Anaesthesia and Perioperative Medicine, Sunshine Coast University Hospital, Birtinya, QLD Australia; 6 School of Pharmacy and Medical Sciences, Griffith University, Southport, QLD Australia; 7 Cardiovascular Research Institute Basel, Basel, Switzerland; 8 Department of Intensive Care, North Estonia Medical Centre, Tallinn, Estonia; 9 School of Mechanical, Medical and Process Engineering, Faculty of Engineering, Queensland University of Technology, Brisbane, QLD Australia; 10 Department of Anaesthesia and Medical Perfusion & Department of Intensive Care, Princess Alexandra Hospital, Brisbane, QLD Australia; 11 Wellcome Trust Centre for Global Health Research, Imperial College London, London, UK; 12 Initiative to Develop African Research Leaders (IdeAL), Kilifi, Kenya; 13 School of Biomedical Sciences, Faculty of Medicine, University of Queensland, Brisbane, QLD Australia; 14 Department of Anaesthesia and Perfusion, The Prince Charles Hospital, Brisbane, QLD Australia; 15 Department of Thoracic and Cardiovascular Surgery, College of Medicine, Korea University, Seoul, Republic of Korea; 16 School of Public Health and Social Work, Faculty of Health, Queensland University of Technology, Brisbane, QLD Australia; 17 School of Veterinary Science, The University of Queensland, Gatton Campus, Brisbane, QLD Australia; 18 Department of Anesthesia, The Alfred Hospital, Melbourne, VIC Australia; 19 School of Pharmacy and Medical Sciences, Griffith University, Southport, Australia; 20 Cardiothoracic Surgery and Transplantation, The Alfred Hospital, Melbourne, VIC Australia; 21 Monash University, Melbourne, VIC Australia; 22 Queensland University of Technology, Brisbane, Australia; 23 Intensive Care Unit, St Andrew’s War Memorial Hospital, Spring Hill, QLD Australia; 24 Wesley Medical Research, Brisbane, Australia; 25 Intensive Care Unit, The Wesley Hospital, Auchenflower, QLD Australia

**Keywords:** Ventilation, Cardiopulmonary bypass, Heart transplant

## Abstract

Open-lung ventilation during cardiopulmonary bypass (CPB) in patients undergoing heart transplantation (HTx) is a potential strategy to mitigate postoperative acute respiratory distress syndrome (ARDS). We utilized an ovine HTx model to investigate whether open-lung ventilation during CPB reduces postoperative lung damage and complications. Eighteen sheep from an ovine HTx model were included, with ventilatory interventions randomly assigned during CPB: the OPENVENT group received low tidal volume (V_T_) of 3 mL/kg and positive end-expiratory pressure (PEEP) of 8 cm H_2_0, while no ventilation was provided in the NOVENT group as per standard of care. The recipient sheep were monitored for 6 h post-surgery. The primary outcome was histological lung damage, scored at the end of the study. Secondary outcomes included pulmonary shunt, driving pressure, hemodynamics and inflammatory lung infiltration. All animals completed the study. The OPENVENT group showed significantly lower histological lung damage versus the NOVENT group (0.22 vs 0.27, *p* = 0.042) and lower pulmonary shunt (19.2 vs 32.1%, *p* = 0.001). In addition, the OPENVENT group exhibited a reduced driving pressure (9.6 cm H_2_O vs. 12.8 cm H_2_O, *p* = 0.039), lower neutrophil (5.25% vs 7.97%, *p* ≤ 0.001) and macrophage infiltrations (11.1% vs 19.6%, *p* < 0.001). No significant differences were observed in hemodynamic parameters. In an ovine model of HTx, open-lung ventilation during CPB significantly reduced lung histological injury and inflammatory infiltration. This highlights the value of an open-lung approach during CPB and emphasizes the need for further clinical evidence to decrease risks of lung injury in HTx patients.

**Supplementary Information:**

The online version contains supplementary material available at 10.1186/s40635-024-00669-w.

## Introduction

Cardiopulmonary bypass (CPB) is an extracorporeal cardio-respiratory support strategy during cardiac surgical procedures. CPB allows the heart to be isolated from the systemic circulation, creating a bloodless surgical field while maintaining control of perfusion, gas exchange and temperature. Despite this support, mild–moderate acute respiratory distress syndrome (ARDS) is a common postoperative complication following cardiac surgery and is associated with increased morbidity and mortality [1, 2].

The pathophysiology of ARDS post CPB is complex and multifactorial [3]. CPB-related systemic inflammatory response syndrome (SIRS) is triggered through exposure of blood to the extracorporeal circuit and contributes to the parenchymal damage and pulmonary capillary dysfunction [4–7]. Localized lung damage can be caused by ischemia during surgery and the subsequent ischemia–reperfusion injury following the restoration of blood flow [8, 9]. Lung deflation is commonly implemented during CPB to optimize the surgical field, and can further amplify local damage leading to atelectasis and decreased alveolar surfactant production [4, 10]. Consequently, following CPB, patients may present with substantial derangements in pulmonary mechanics and gas exchange which lead to ARDS [11, 12].

Patients requiring heart transplantation (HTx) represent an especially vulnerable surgical cohort. The prolonged use of CPB, transplanted heart function, and intraoperative long-term immunosuppression all contribute to a significantly increased risk of postoperative pulmonary complications [1, 2, 13]. The risk of ARDS associated with HTx surgery could be mitigated by preventing lung deflation, through implementation of open-lung ventilation strategies during CPB [14]. The combination of positive end-expiratory pressure (PEEP) and low tidal volume (V_T_) optimizes alveolar recruitment, while minimizing lung movement and ventilator-induced lung injury (VILI). Recent animal and human studies have demonstrated that continuation of open-lung ventilation through CPB can improve gas exchange, while reducing inflammation, endothelial dysfunction and the occurrence of postoperative complications [11, 15–22]. Despite this promising evidence, the recent large-scale PROVECS and MECANO trials reported no improvement in postoperative pulmonary complications, after the implementation of an open-lung ventilation strategy [23, 24]. While these trials recruited patients undergoing a variety of cardiac surgical procedures, patients undergoing HTx procedures were not included. The evidence regarding the effectiveness of open-lung ventilation during CPB to reduce postoperative pulmonary complications is mixed, and there is a paucity of data regarding its specific use during HTx surgery.

We have previously developed a clinically relevant sheep model of orthotopic HTx to investigate the application of hypothermic oxygenated perfusion (HOPE) to donor heart preservation [25, 26]. During the HOPE preservation study, we simultaneously investigated the application of open-lung ventilation during CPB, hypothesizing that it could further reduce postoperative pulmonary complications. We performed a single-blinded, randomized controlled trial, using an ovine HTx model to confirm that open-lung ventilation could mitigate histologically confirmed postoperative lung damage and decrease short-term postoperative pulmonary complications.

## Methods

### Animals and ethics

An ovine HTx model was performed using pairs of matched female sheep. In this model, donor sheep were either brain stem death (BD) or brain stem viable (sham) and monitored for 24 h prior to cardiac surgery. After heart excision, the donor heart was preserved in either static cold storage (SCS) for 2 h or HOPE for 2 or 8 h. Further details regarding the HTx model and the novel HOPE methods have been previously reported [26].

Eighteen recipient sheep from the ovine HTx model were included in our study. Animals reported in this study represent a subset of those presented in the previously published HOPE trial [25, 26]. All recipient animals were placed on CPB during surgery, and all surgical procedures were performed by qualified personnel. The project was approved by Queensland University of Technology (QUT) Animal Ethics Committee (Approval #16–1109). Ratified by the University of Queensland AEC (QUT/393/17/QUT), experiments were performed in accordance with the National Health and Medical Research Council (NHMRC) Australian Code of Practice for the Care and Use of Animals for Scientific Purposes (8th Edition 2013), the Animal Care and Protection Act 2001 (QLD) and complied with the ARRIVE Guidelines.

### Anesthesia and surgery

**Fig. 1:**
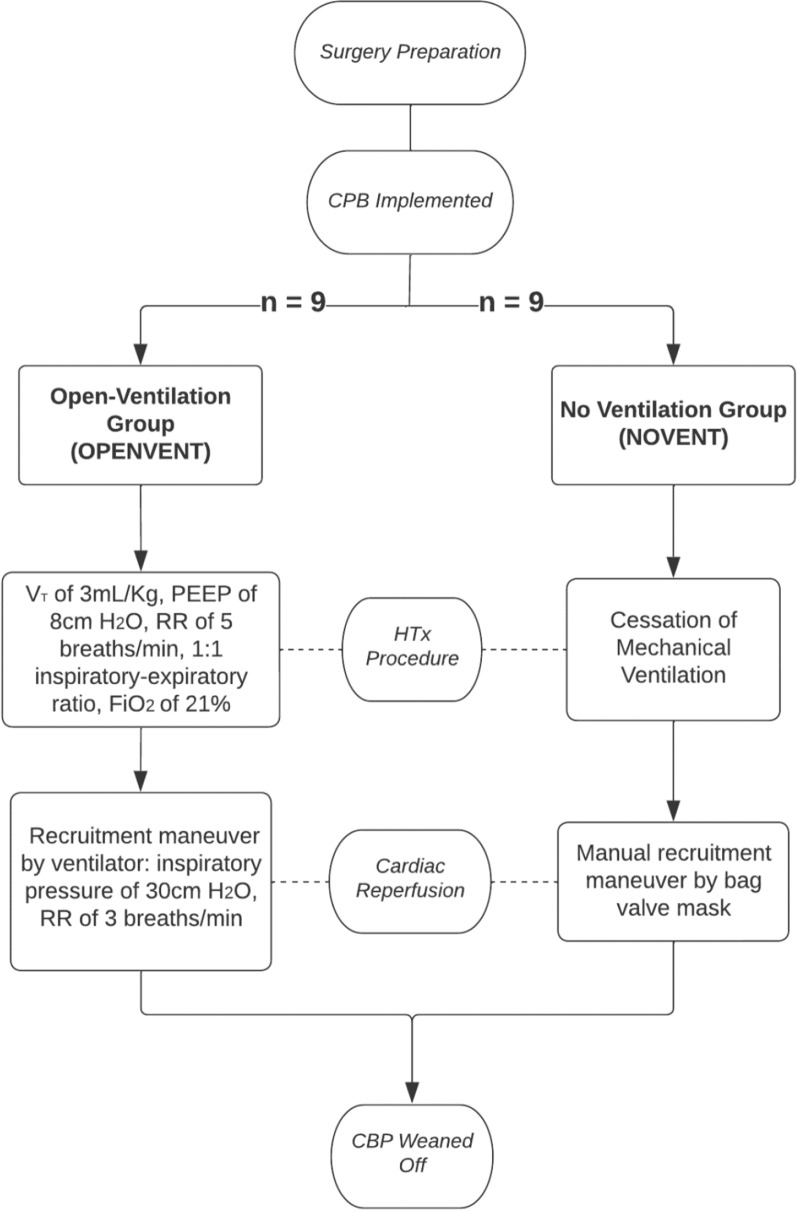
Flowchart describing experimental groups (*RR* respiratory rate, *V*_*T*_ tidal volume, *PEEP* positive end-expiratory pressure, *HTx* heart transplant). Created with LucidCharts.com

Electrocardiography (ECG), pulse oximetry, and capnography were monitored throughout the study. Anesthesia in recipient sheep was induced by intravenous propofol injection (3–4 mg/kg), followed by orotracheal intubation and placement on a surgical table in the supine position. Anesthesia was maintained with continuous intravenous infusions of fentanyl (5-15 µg/kg/h), midazolam (0.5–0.8 mg/kg/h), and ketamine (2.5–7.5 mg/kg/h). Initially, the animals were mechanically ventilated with a V_T_ of 8 ml/kg and PEEP of 5 cm H_2_O to achieve arterial oxygen saturation of 92% [27, 28]. Respiratory rate (RR) and fraction of inspired oxygen (FiO_2_) were further titrated to normalize pH, arterial partial pressure of carbon dioxide (PaCO_2_) and arterial partial pressure of oxygen (PaO_2_). A Swan–Ganz catheter was advanced from the right jugular vein to the pulmonary artery to continuously measure cardiac output and mixed venous oxygen saturation. Pulmonary artery pressure and pulmonary vascular resistance were recorded throughout the study. Following administration of vecuronium (0.1 mg/kg IV) and optimization of anesthesia, a median sternotomy was performed. Heparin was administered (100–300 U/kg) to achieve an activated clotting time > 400 s. After cannulation of the inferior vena cava, superior vena cava and aortic arch, CPB was commenced. The target flow rate was 50–60 ml/kg to achieve a mean arterial pressure of > 60 mmHg. Once CPB was stabilized within these parameters, the aorta was cross-clamped and both vena cavae were snared allowing excision of the native heart. The donor heart, from another cross-matched, immune-compatible sheep was then orthotopically transplanted. Using pentobarbitone (0.5 mL/kg), both donor and recipient animals were humanely euthanized at completion of study. During CPB and the transplant procedure in the recipient sheep, ventilatory interventions were applied to the sheep according to the randomly assigned experimental groups (Fig. 1).

- The Open-Lung Ventilation Group (OPENVENT) was ventilated from the commencement of CPB with a V_T_ = 3 mL/kg, PEEP = 8 cm H_2_O, RR = 5 breaths/min, 21% FiO_2,_ and 1:1 inspiratory/expiratory ratio [23, 24]. These settings ensured minimal disturbance to the surgical field, while allowing the lungs to remain open during CPB. During donor heart reperfusion, the ventilator was used to perform recruitment maneuvers with an inspiratory pressure of 30 cm H_2_O and RR of 3 breaths/min. Inspiratory/expiratory ratio of 1:1 was maintained during the procedure.
- The No Ventilation Group (NOVENT) received no ventilatory intervention during CPB, resulting in lung deflation, and no airway pressure monitoring was applied. During cardiac reperfusion, recruitment maneuvers were performed manually with a bag valve mask connected to the endotracheal tube and with PEEP valve set at 7.5 cm H_2_O. Notably, as in clinical practice, airway pressure was not monitored during the recruitment phase. Instead, we ensured lung re-expansion by observing the surgical field.

Upon completion of the anastomoses, the animals were rewarmed using CPB and methylprednisolone (250 mg) was administered intravenously. The aortic cross clamp was removed to reperfuse the cardiac allograft. The heart was rested for 30 min, with inotropic, chronotropic, and vasopressor supports administered as dictated by echocardiographic and hemodynamic cardiac function. Following weaning from CPB, protamine was administered and mechanical ventilation resumed in both groups during the postoperative monitoring period with the following settings: V_T_ = 6 mL/kg, RR = 12–20 breaths/min to achieve PaCO_2_ of 36-44 mmHg, 1:2 inspiratory/expiratory ratio, and PEEP/FiO_2_ adjusted to achieve at least PaO_2_ = 100 mmHg [29, 30]. Lung-protective ventilation with abovementioned settings were applied to reduce risk of VILI in the postoperative period. The sternotomy wound and chest wall was left open during the postoperative period. Monitoring and management of recipient sheep continued for 6 h following separation from CPB, with regular assessment of pulmonary and cardiac functions. Animals were humanely euthanized at the end of the 6-h monitoring period, which was determined by the orthotopic HTx and HOPE preservation main study.

### Sampling and analyses

Data were collected from preoperative baseline recordings (BSL) and hourly in the 6-h postoperative monitoring period following completion of transplant: T0 (successful weaning from CPB), T0.5, T1, T2, T3, T4, T6 (6 h post-transplant). Arterial and mixed venous blood gases, ventilation and hemodynamics were recorded for all timepoints. Blood gas analyses were recorded using the ABL800 Flex Blood Gas Analyzer (Radiometer). Full blood counts and biochemistry of whole blood (EDTA) and plasma samples were assessed externally (IDEXX Laboratories, Brisbane, Australia). Hematological profiles and blood biochemistry were analyzed on a Sysmex XT2000i-V hematology analyzer, and a Beckman Coulter AU680 ISE chemistry analyzer, respectively. A PowerLab data acquisition system (model ML880) was utilized in conjunction with Labchart 7 (AD Instruments, Bella Vista, Australia) to record hemodynamics. Continuous cardiac output, mean arterial pressure, heart rate, end tidal carbon dioxide (ETCO_2_), oxygen saturation (SPO_2_) and mixed venous oxygen saturation (S_V_O^2^) were continuously measured through both the Vigilance II Monitor (Edward Lifesciences CA, USA) and the Marquette Solar 8000 (GE Healthcare ILL USA). Following sheep euthanasia, the lungs were excised and dissected. Histological and frozen samples were taken from each lobe for further analysis (right upper lobe (RUL), right middle lobe (RML), right lower lobe (RLL), left upper lobe (LUL), left lower lobe (LLL)).

### Outcomes

#### Primary outcome

The primary outcome was histological lung damage. Tissue samples for histological analysis were dissected immediately after excision of lungs from euthanized recipient sheep and fixed in 10% buffered formalin for 24 h. 5 samples were taken from each lobe. Processed samples were embedded in paraffin, sectioned to 5 μm thickness and stained with hematoxylin and eosin (H&E). A Brightfield light microscope was used by a single senior veterinary pathologist blinded to treatment allocation to examine histological lung damage, using a modified scoring system from the American Thoracic Society [31]. Macroscopically, examination and sampling were targeted to the most damaged regions. Eleven criteria were utilized in a scoring system adapted from Kulkarni et al. [31], which assessed the debris in airspaces, alveolar epithelial injury and thickening, presence of neutrophils, thrombi, capillary damage, atelectasis and septal muscle hypertrophy. The scoring system is shown in greater detail in Table E2 (Supplementary Material). Lung slides were scored from 0 to 2 dependent on their features, and 10 lung fields were assessed to produce a mean damage score for each lobe.

#### Secondary outcomes

Secondary outcomes comprised several postoperative pulmonary complications, which can be broadly defined as any complication involving the respiratory system occurring after surgery. Pulmonary inflammation was determined through quantification of neutrophil and macrophage infiltration, using immunohistochemistry (IHC) and ImageJ. ImageJ is a widely used Java-based image processing program that was developed by the National Institutes of Health, Bethesda, MD, USA. It is an open-source software, and facilitates the visualization, inspection and quantification of scientific image data [32–35]. Images were collected using the AxioImager.Z1 motorized upright microscope (Carl Zeiss) and visualized using ZEN 2.0 (blue edition) (Carl Zeiss). In-house enzyme linked immunosorbent assays (ELISAs) were performed to detect interleukin 8 (IL-8) per previously published protocols [36]. Ventilatory parameters were recorded via the Te Hamilton-G5 ventilator at 100 Hz, and functional measurements of gas exchange and pulmonary mechanics were calculated. Epicardial echocardiography was performed in recipients at baseline (BSL), and during the post-monitoring period at T0, T1, T3 and T6 (completion of transplant). An X5-1 transducer with a spacer connected to an IE-33 ultrasound scanner (Philips, Bothell, WA, USA) was used for image obtainment. Analysis was performed to collect end-diastolic area (EDA), end-systolic area (ESA), fractional area change (FAC), endo-myocardial global circumferential strain (EndoGCS) and global radial strain (GRS). Further data were collected regarding hemodynamics, oxygen delivery/consumption, arterial lactate, and base excess. Extended description of the methods and calculations used to measure the secondary outcomes can be found in the Supplementary Material.

### Statistical analysis

This study was conducted alongside the primary study; thus, a predetermined sample size was not calculated. Baseline characteristics were summarized using means and standard errors for normally distributed data, and medians with interquartile ranges for non-normally distributed data. Between-group differences in baseline characteristics were compared using unpaired t-tests and Mann–Whitney tests. We performed a mixed-effects analysis using the PROC MIXED procedure in SAS to assess the effects of OPENVENT and NOVENT on the histology score, while accounting for the repeated measures across pulmonary lobes. A variance components covariance structure was applied to model the random effects. For multiple pairwise comparisons among lobes, Sidak's adjustment was utilized to control the family-wise error rate. Two-way ANOVA was used to analyze secondary outcomes of neutrophil and macrophage lung tissue infiltration. Ventilation strategy and lobe were the two categorical variables, with NOVENT set as reference level for strategy. A two-way interaction term was also specified. Secondary outcomes including gas exchange, pulmonary mechanics, hemodynamic parameters and epicardial echocardiography were analyzed by linear mixed modeling. Ventilation strategy and time point were included in the model as categorical fixed effects, with NOVENT and BSL set as reference levels for strategy and time points, respectively. A two-way interaction term was also specified. A random effect was specified per sheep to account for repeated measurements. Post hoc multiple comparisons were performed for primary and secondary endpoints in cases of a statistically significant interaction term. Sidak’s correction was applied to adjust the family-wise error rate. Residual assumptions were examined through quantile–quantile (QQ) plots, to inform appropriate outcome transformations. Data for respiratory system compliance was non-normally distributed, and natural log transformation was applied. Statistical significance was defined as a p-value less than 0.05. Analysis was performed utilizing SAS 9.4 (SAS Institute Inc., Cary, NC, USA.) and Graphpad Prism 9.0 (GraphPad Software, Boston, Massachusetts USA). Finally, a post hoc power calculation was performed for the primary outcome of the study by simulating from the fitted mixed model, with and without ventilation strategy included as a fixed effect. This simulation therefore tested the hypothesis of no difference between ventilation strategies. The simulation was completed using the simr R package in R version 4.3.3 and it is reported in the supplementary results.

## Results

### Population

Eighteen experiments were completed, nine of those were enrolled into the OPENVENT group and nine into the NOVENT group. The donor hearts were retrieved from sham or BD donors (BD: 9 experiments, Sham: 9 experiments), and the preservation method varied. The allocation of preservation method was randomized between intervention groups. The NOVENT group contained 3 × 2 h HOPE, 5 × 8 h HOPE and 1 SCS while the OPENVENT group contained 3 × 2 h HOPE, 4 × 8 h HOPE and 2 SCS (Table E1, Supplementary Material). There were no statistical differences between recipient groups in preoperative clinical measurements, intraoperative characteristics, or baseline respiratory parameters (Table 1).

**Table 1:** Data are presented as mean ± SE and median (IQR)

	NOVENT group (n = 9)	OPENVENT group (n = 9)	P value
Preoperative characteristics			
Weight (kg)	52.0 ± 2.62 *52 (9.5)*	51.1 ± 1.29 *51 (4.5)*	0.987
Temperature (°C) *	38.3 ± 0.29 *38.6 (0.98)*	38.8 ± 0.12 *38.8 (0.4)*	0.436
Mean arterial pressure (mmHg)	101 ± 3.71 *105 (18)*	94.6 ± 3.74 *98 (18)*	0.901
Heart rate (bpm)	86.3 ± 8.46 *82.5 (38.8)*	91.8 ± 7.86 *87 (31.5)*	0.921
Surgical characteristics			
CPB time (mins)	180 ± 5.95 *183 (21.8)*	190 ± 3.97 *190 (15)*	0.905
Aortic cross clamp time (mins)	73.5 ± 10.2 *65 (33)*	63.7 ± 1.86 *65 (6)*	0.625
Blood transfusions (mL)	181.9 ± 91.9 *0 (500)*	211 ± 151 *0 (300)*	0.592
Baseline respiratory parameters			
PaCO_2_ (mmHg)	39.3 ± 1.3 *38.8 (5.85)*	45.2 ± 3.61 *41.3 (7.15)*	0.914
Driving pressure (cm H_2_O)	11.3 ± 2.77 *11.5 (15.8)*	7.87 ± 2.82 *9 (8)*	0.954
A-a gradient (mmHg)	90.1 ± 23.5 *77 (77.2)*	90.6 ± 25.1 *65.5 (140)*	0.993
PaO_2_/FiO_2_ (mmHg)	439 ± 47.4 *462 (169)*	435 ± 44.3 *492 (255)*	0.939
Respiratory system compliance (mL/cm H_2_O)*	33.1 ± 3.71 *30.9 (21.9)*	47.46 ± 14.4 *36.9 (36.1)*	0.645
Shunt (%)	18.7 ± 2.71 *14.7 (11.7)*	19.9 ± 1.22 *20.3 (4.58)*	0.987
Physiological dead space (%)	9.53 ± 4.19 *7.2 (21.7)*	6.01 ± 7.66 *− 0.38 (40.3)*	0.951
Minute volume (L/min)	6.00 ± 0.314 *5.9 (1.72)*	6.70 ± 0.151 *6.59 (0.88)*	0.990

*Non-normally distributed data. CPB, cardiopulmonary bypass; PaCO_2_, arterial partial pressure of carbon dioxide; A-a, alveolar–arterial; PaO_2_/FiO_2_, ratio between arterial partial pressure of oxygen and inspiratory fraction of oxygen

### Primary outcome

**Fig. 2:**
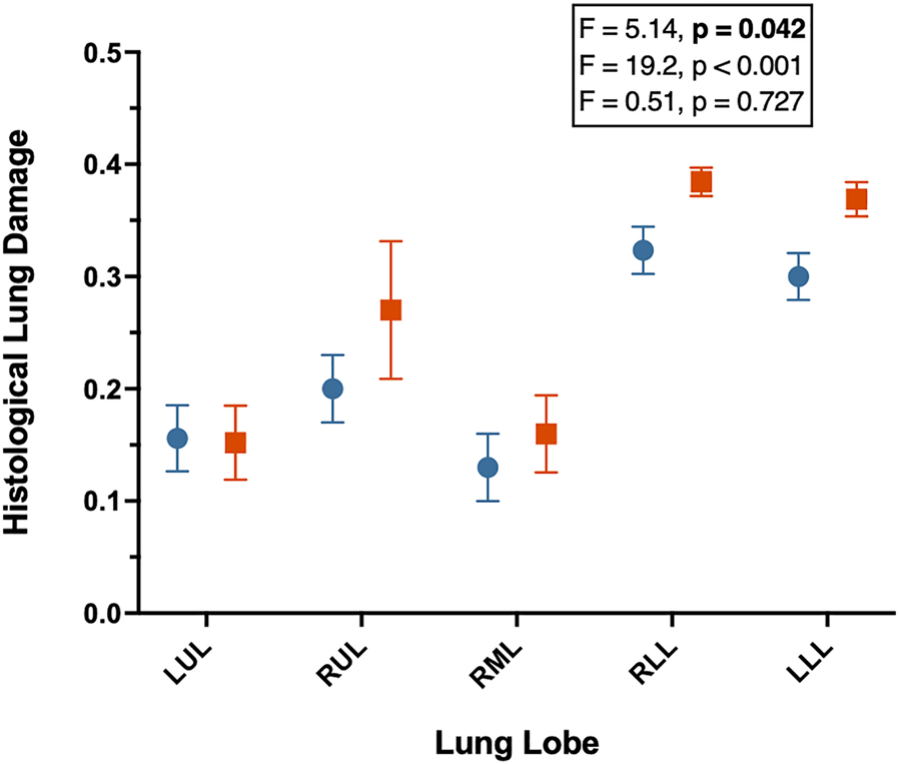
**A** Histological Lung Injury Score from recipient sheep treated with either no ventilation (NOVENT ■) or open-lung ventilation (OPENVENT ●) during cardiopulmonary bypass. Data were clustered into lobes and shown as mean ± SEM with *n* = 8 for both groups. The effect size and *p*-values of the mixed analysis are reported from top to bottom of factor ventilatory strategy, lobe and interaction between these two factors. *LUL* left upper lobe, *RUL* right upper lobe, *RML* right middle lobe, *RLL* right lower lobe and *LLL* left lower lobe

**Fig. 3:**
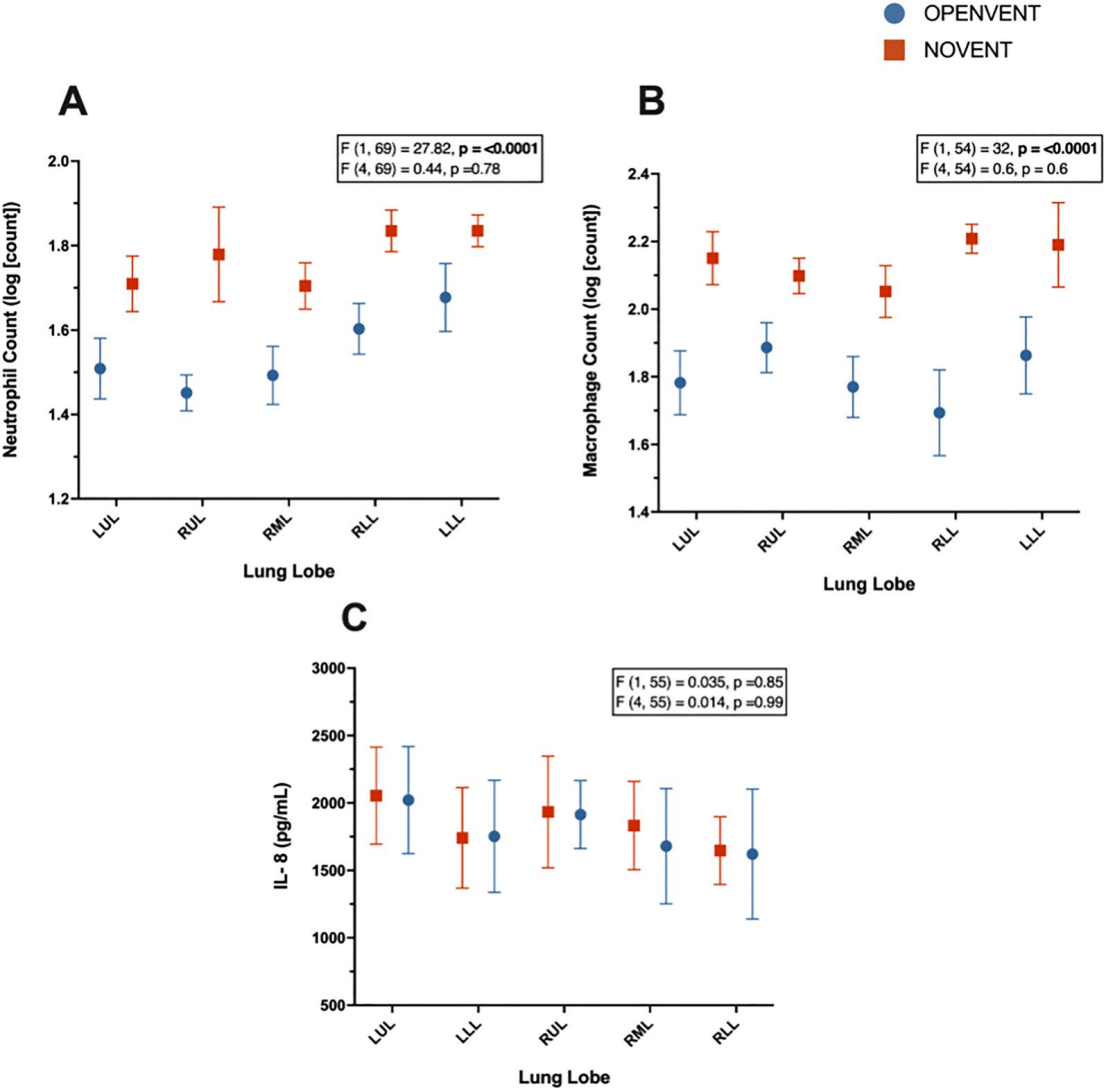
**A** Neutrophil counts (log [count]), **B** macrophage counts (log [count]), **C** interleukin-8 (pg/mL) concentrations in postoperative lung samples from recipient sheep treated with either no ventilation (NOVENT ■) or open-lung ventilation (OPENVENT ●) during cardiopulmonary bypass. Data were segmented into lobes, and shown as mean ± SEM with n = 8 for both groups. *F* (a, b) = c, *a* represents the between group variance, *b* the within-group variance, the F value (*c*) is the ratio of the variation between sample means/ variation within samples. Top F and p values refer to effect of ventilation strategy on outcome, and bottom F and p values refer to the combined effects of ventilation strategies and lung lobe (interaction term). *LUL* left upper lobe, *RUL* right upper lobe, *RML* right middle lobe, *RLL* right lower lobe and *LLL* left lower lobe

Atelectasis, interstitial infiltration by neutrophils, thickening of septa, muscle hypertrophy were the most common histological features of injury. Overall, the OPENVENT group showed a significantly lower histological lung damage score when compared to the NOVENT group (0.221 vs 0.267; F = 5.14, p = 0.042). Among all tested lobes, right and left lower lobes showed higher injury score (F = 19.2, p < 0.001). See Fig. 2 for details, and Fig. 3 for histological images.

### Secondary outcomes

#### Pulmonary inflammation

**Fig. 4:**
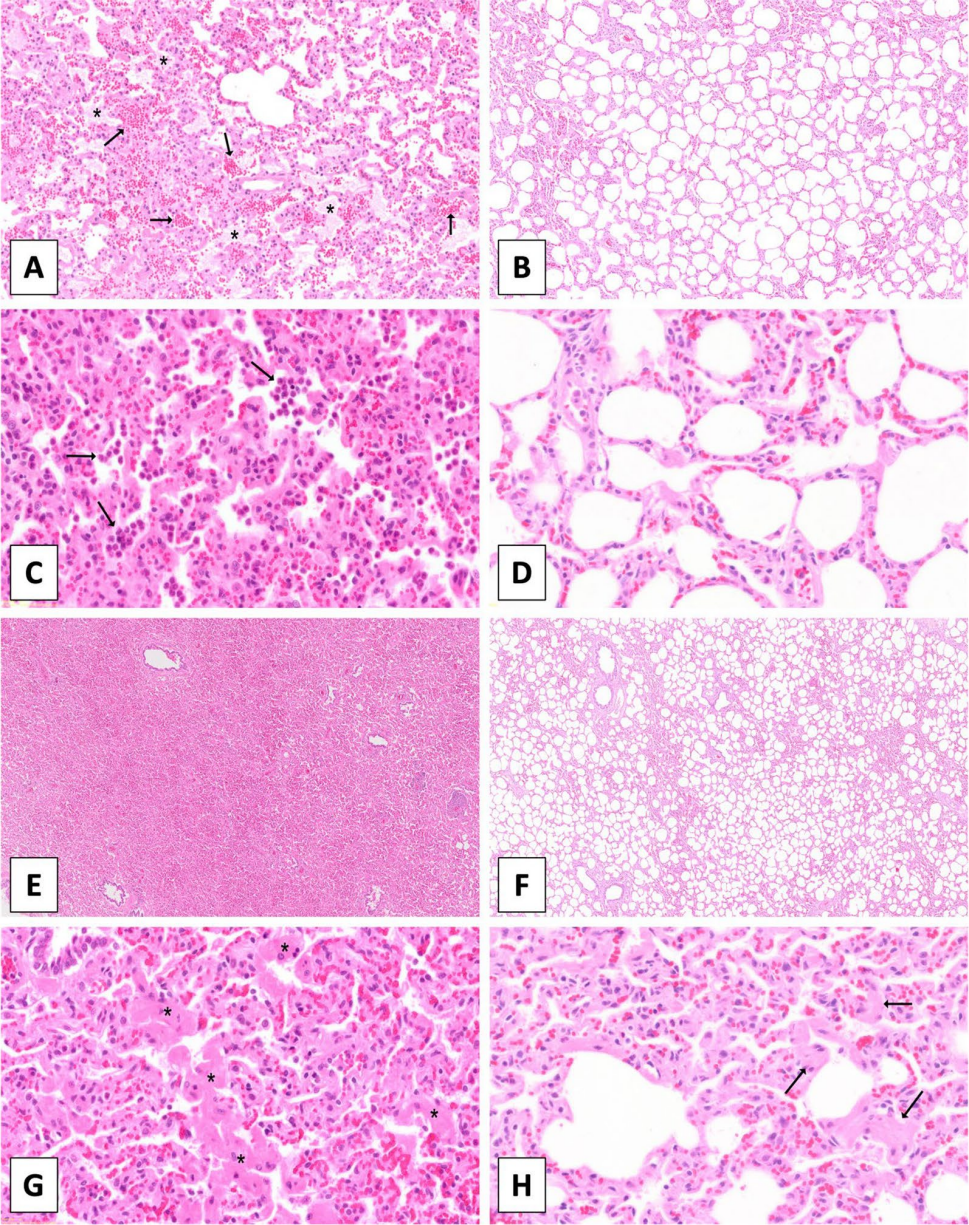
Histology images (H&E staining), demonstrating typical histological features. **A** Presence of mild intra-alveolar edema (asterisks) and hemorrhage (arrows). **B** Absence of edema or intra-alveolar hemorrhagic material. **C** Intra-alveolar accumulation of neutrophils (arrows) and mild increase in the numbers of interstitial neutrophils. **D** Low numbers of neutrophils within the interstitial space. **E** Severe diffuse atelectasis with alveolar collapse. **F** Normally aerated alveoli. **G** Severe multifocal thickening of the alveolar septa caused by hypertrophy of smooth muscle cells (asterisks). (H) Mild septal smooth muscle hypertrophy (asterisks). **A**, **B** Magnification 5x. **C**, **D**, **G**, **H** Magnification 20x. **E**, **F** Magnification 2x

**Fig. 5:**
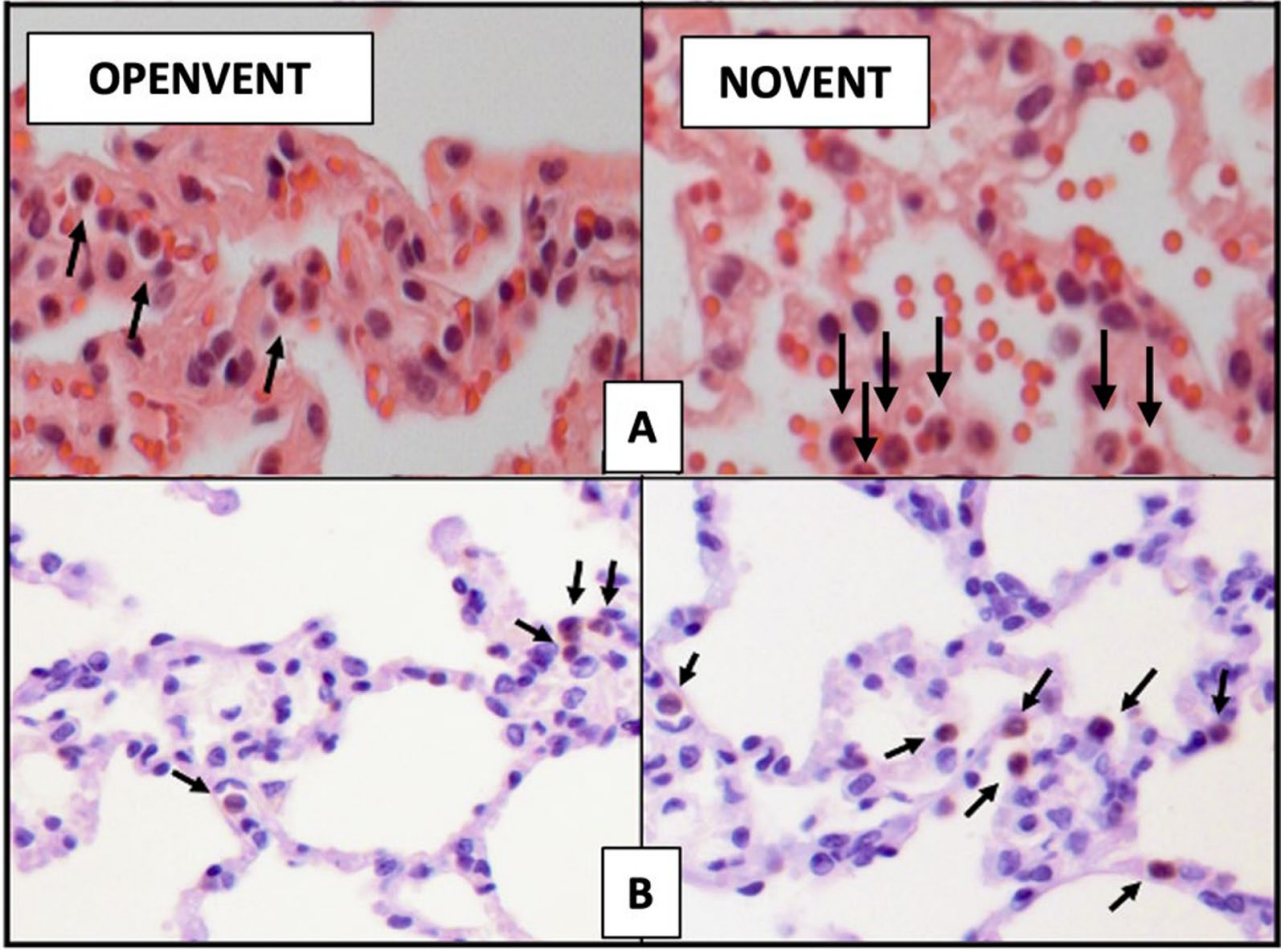
**A** Histology images (H&E staining) used for neutrophil quantification in tissue samples. Brightfield light microscope used to capture images, with arrows indicating neutrophils. Magnification 40X. **B** Images from IHC staining used for macrophage quantification in postoperative samples. Brightfield light microscope used to capture images, with arrows indicating macrophages. Magnification 40X

The OPENVENT group presented a significantly lower neutrophil count in the lung tissue (reported as the log of the absolute number of neutrophils), in comparison to the NOVENT group (1.55 vs 1.77; difference between groups = − 0.23, 95% CI [-0.31 to -0.14]; F (1, 69) = 27.8, p ≤0.001). Similarly, the OPENVENT group contained a significantly lower macrophage count in the lung tissue (reported as log of absolute number of macrophages) in comparison to the NOVENT group (1.78 vs 2.14; difference between groups = − 0.34, 95% CI [− 0.46 to -0.22]; F (1, 54) = 32, p < 0.001). Finally, no difference in IL-8 concentration was found between ventilation groups. See Fig. 4 for details and Fig. 5 for histological images.

#### Gas exchange

**Fig. 6:**
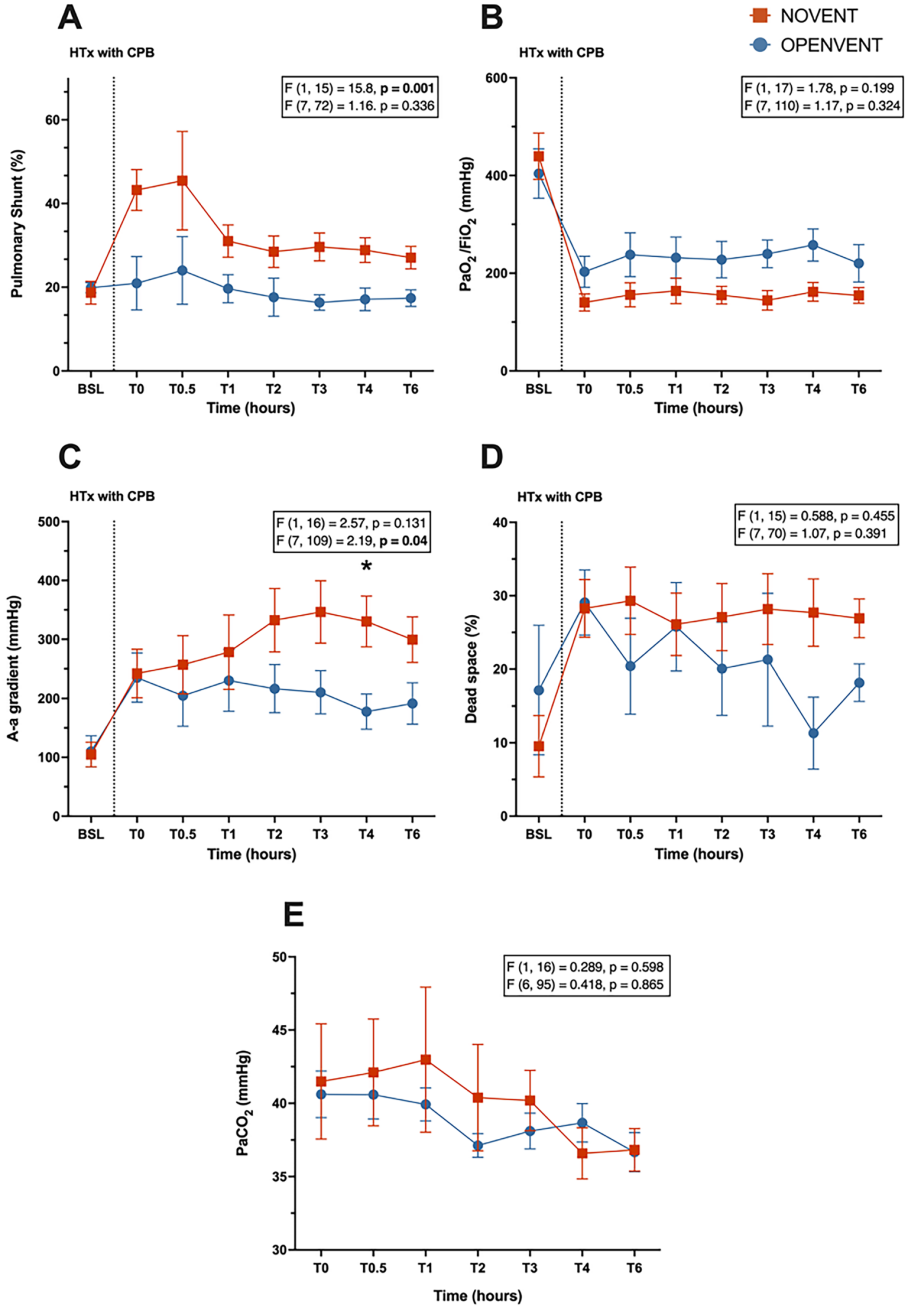
**A** Pulmonary shunt (%), **B** PaO_2_/FiO_2_ ratio (mmHg), **C** A-a gradient (mmHg), **D** physiological dead space (%), **E** PaCO_2_ (mmHg) during 6-h post-transplantation monitoring period in recipient sheep treated with either no ventilation (NOVENT ■) or open-lung ventilation (OPENVENT ●) during cardiopulmonary bypass. Data shown as mean ± SEM from preoperative baseline (BSL) through to 6 h postoperative (T6), with *n* = 9 for both groups. *F* (a, b) = c, *a* represents the between group variance, *b* the within-group variance, the *F* value (*c*) is the ratio of the variation between sample means/ variation within samples. Top *F* and *p* values refer to effect of ventilation strategy on outcome, and bottom *F* and *p* values refer to the combined effects of ventilation strategies and time (interaction term)

The OPENVENT ventilation strategy was associated with a reduced pulmonary shunt (32.1% vs 19.2%; difference between groups = 12.9%, 95% CI [5.97% to 19.8%]; F (1, 15) = 15.8, *p* = 0.001). The OPENVENT group resulted in an improved A-a gradient over time (interaction term: F (7, 109) = 2.19, *p* = 0.040). A statistically significant difference for the A-a gradient was found between ventilation groups at T4 (334 mmHg vs 178 mmHg; Difference between groups at T4 = 173 ± 52.4 mmHg; *p* = 0.045). No significant differences between ventilation groups were found for PaO_2_/FiO_2_ (PF) ratio, PaCO_2_ or physiological dead space ventilation (Fig. 6).

#### Pulmonary mechanics and hemodynamic parameters

**Fig. 7:**
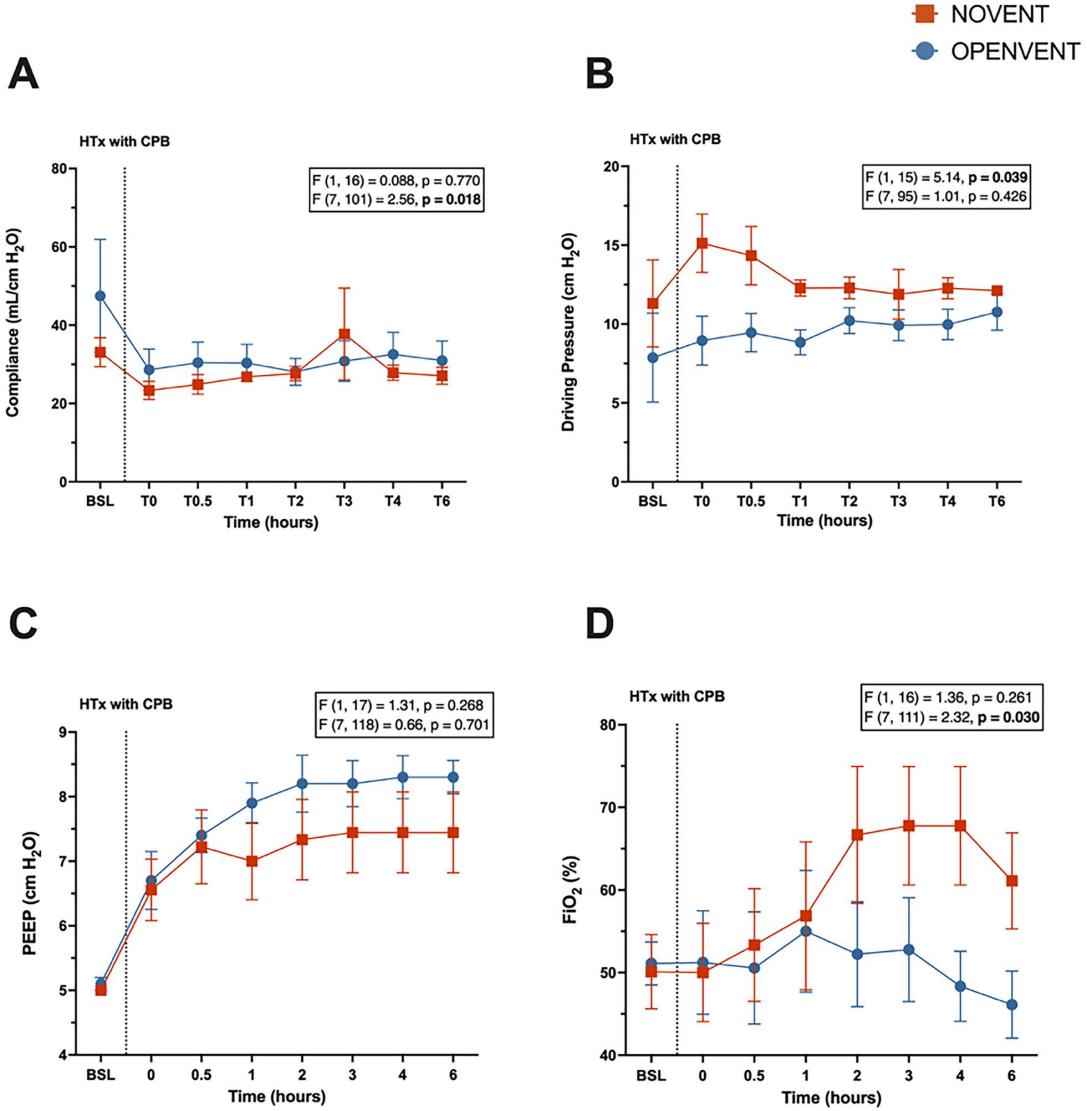
**A** Compliance (mL/ cm H_2_O), **B** driving pressure (cm H_2_O), **C** positive end-expiratory pressure (PEEP) (cm H_2_O), **D** FiO_2_ (%) during 6-h post-transplantation monitoring period in recipient sheep treated with either no ventilation (NOVENT ■) or open-lung ventilation (OPENVENT ●) during cardiopulmonary bypass. Data shown as mean ± SEM from preoperative baseline (BSL) through to 6 h postoperative (T6), with n = 9 for both groups. *F* (a, b) = c, *a* represents the between group variance, *b* the within-group variance, the *F* value (*c*) is the ratio of the variation between sample means/ variation within samples. Top *F* and *p* values refer to effect of ventilation strategy on outcome, and bottom *F* and *p* values refer to the combined effects of ventilation strategies and time (interaction term)

**Fig. 8:**
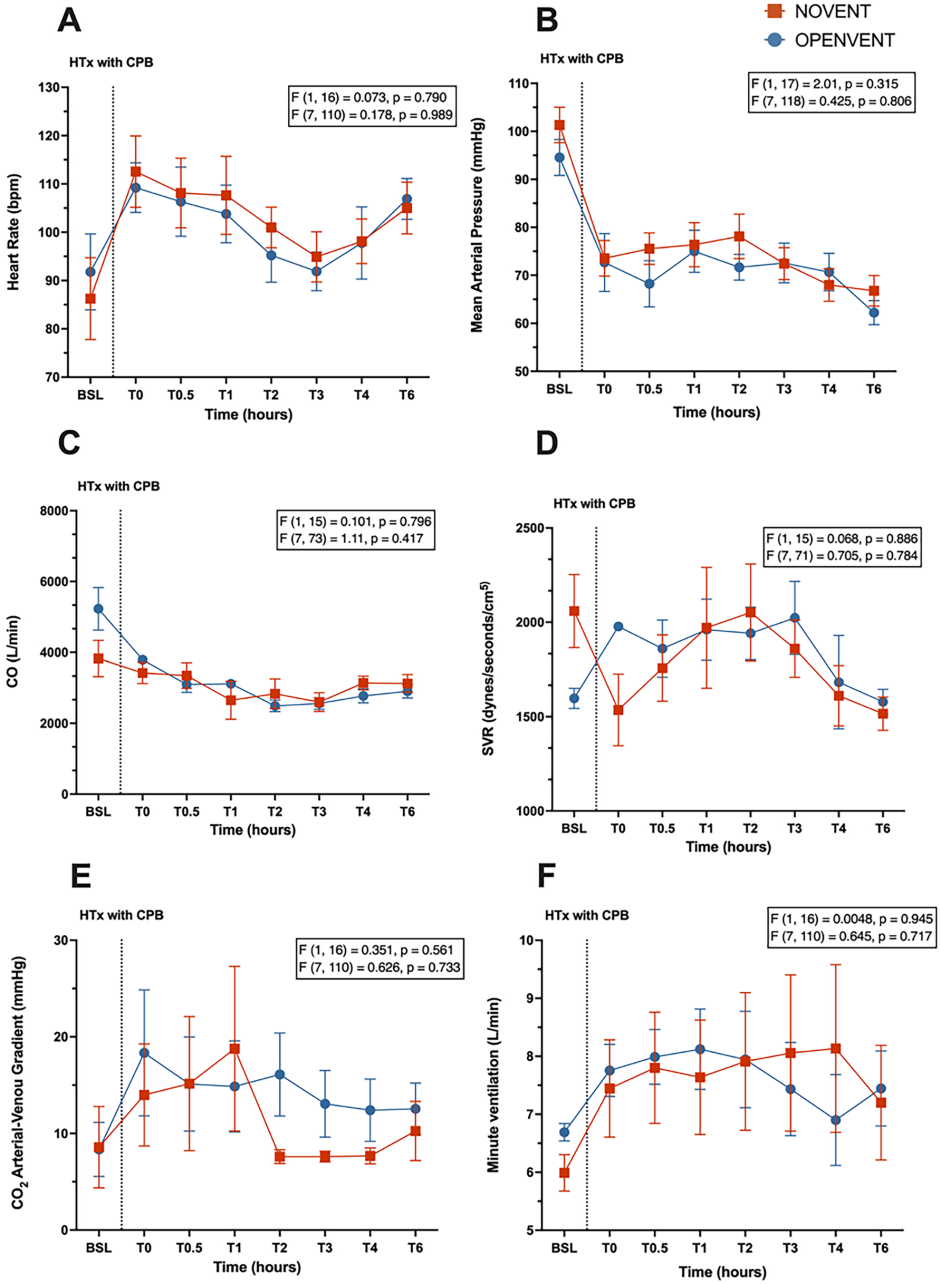
**A** Heart rate (bpm), **B** mean arterial pressure (mmHg), **C** cardiac output (L/min), **D** systemic vascular resistance (dynes/second/cm^5^) and **E** CO_2_ arterial–venous gradient (mmHg), **F** minute ventilation (L/min) during 6-h post-transplantation monitoring period in recipient sheep treated with either no ventilation (NOVENT ■) or open-lung ventilation (OPENVENT ●) during cardiopulmonary bypass. Data shown as mean ± SEM from preoperative baseline (BSL) through to 6 h postoperative (T6), with n = 9 for both groups. *F* (a, b) = c, *a* represents the between group variance, *b* the within-group variance, the F value (*c*) is the ratio of the variation between sample means/ variation within samples. Top *F* and *p* values refer to effect of ventilation strategy on outcome, and bottom F and p values refer to the combined effects of ventilation strategies and time (interaction term)

A lower driving pressure was seen in the OPENVENT strategy group (12.8 cm H_2_O vs 9.64 cm H_2_O; difference between groups = 3.17 cm H_2_O, 95% CI [0.188 cm H_2_O to 6.15 cm H_2_O]; *p* = 0.039). While the interaction term was significant (F (7, 101) = 2.56, *p* = 0.018), the respiratory system compliance did not differ between groups at any timepoint (Fig. 7). Similarly, the interaction term for postoperative FiO_2_ was found to be significant (F (7, 111) = 2.32, *p* = 0.030), but no significant changes were found between groups at any timepoints despite a trend for increased FiO_2_ in the NOVENT group. No significant differences between timepoints were seen in PEEP (Fig. 7), hemodynamic parameters (Fig. 8), ventilatory settings, oxygen delivery and consumption, lactate and acid–base status (Figure E1 and E2 Supplementary Material).

#### Epicardial echocardiography

**Fig. 9:**
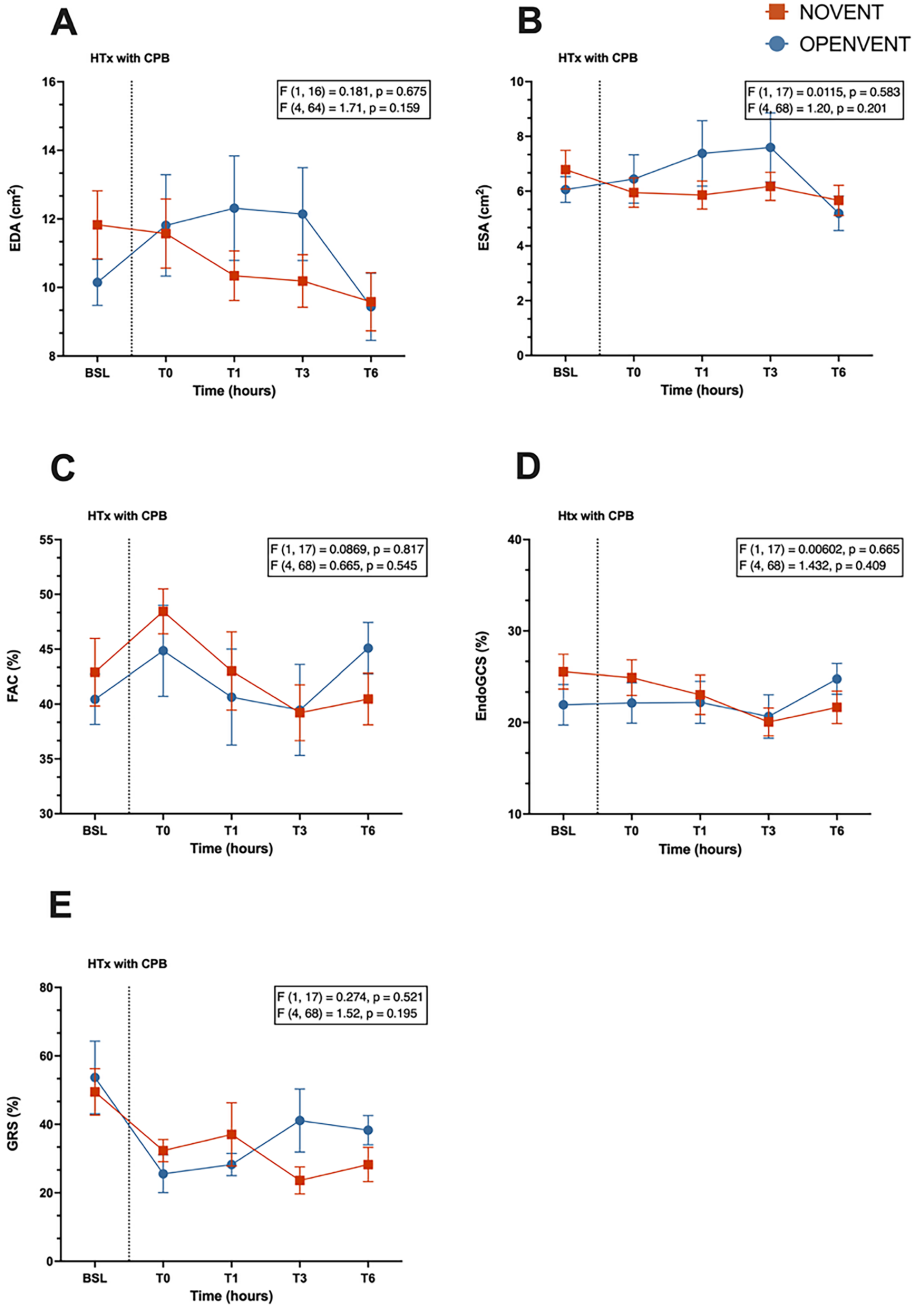
**A** End-diastolic area (EDA, cm H_2_O), **B** end-systolic area (ESA, cm H_2_O), **C** fractional area change (FAC, %), **D** endo-myocardial global circumferential strain (EndoGCS, %) and **E** global radial strain (GRS, %) during 6-h post-transplantation monitoring period in recipient sheep treated with either no ventilation (NOVENT ■) or open-lung ventilation (OPENVENT ●) during cardiopulmonary bypass. Data shown as mean ± SEM from preoperative baseline (BSL) through to 6 h postoperative (T6), with n = 9 for both groups. *F* (a, b) = c, *a* represents the between group variance, *b* the within-group variance, the *F* value (*c*) is the ratio of the variation between sample means/ variation within samples. Top *F* and *p* values refer to effect of ventilation strategy on outcome, and bottom *F* and *p* values refer to the combined effects of ventilation strategies and time (interaction term)

No significant differences were seen in any measurements of heart function and contractility (Fig. 9).

## Discussion

In this ovine HTx model, open-lung ventilation throughout CPB demonstrated reduction in lung histological injury. Improvements in other secondary endpoints were also observed in the open-lung ventilation group, including pulmonary shunt, A-a gradient, inflammatory cell infiltration, and lung mechanics.

Our findings indicate an improved pulmonary shunt in the OPENVENT group, resulting in a reduction of postoperative lung dysfunction and ventilation/perfusion (V/Q) mismatching. Lung deflation during CPB causes significant regional atelectasis and pulmonary edema, with resumption of ventilation seldom leading to complete lung re-expansion [37]. Maintaining ventilation during CPB has been demonstrated by Kirmani and colleagues to prevent perioperative deflation, and significantly decrease atelectasis after surgery in patients undergoing coronary artery bypass grafts (CABG) [38]. Multiple trials have concluded that residual atelectasis significantly contributes to deterioration in postoperative oxygenation measurements, such as A-a gradient and pulmonary shunt [39, 40]. Our findings corroborate those made by Magnusson and colleagues, as their porcine model demonstrated an increase in shunt fraction after CPB with lung deflation, strongly correlating with atelectasis [11]. Further, the link between the increased shunt fraction and postoperative atelectasis is strengthened by our findings of histological damage in the lower lobes of the NOVENT group. This suggests an increase in alveolar collapse, as histology scoring primarily assessed the presence of atelectasis, along with other vascular, bronchiolar, and extravascular aspects. Since the samples were taken at the end of the monitoring period, we cannot determine whether the atelectasis occurred primarily due to the CPB-related deflation or developed at a later stage. Previous data from D’Angelo and colleagues suggest a combination of both likely occurred in these settings, indicating that alveolar collapse associated with perioperative deflation caused epithelial damage, impairing surfactant secretion and subsequently increasing the likelihood of further atelectasis at later stages [41]. Regarding the increased damage observed in the lower lobes, explanations for this phenomenon can only be speculative. It is plausible that the prominent atelectasis, induced by diaphragmatic splinting might contribute to this effect. Additionally, recruitment and overdistension could be another factor influencing the higher damage observed in the lower lobes.

Maintaining ventilation through CPB appeared to mitigate respiratory dysfunction, with the OPENVENT group exhibiting significantly lower A-a gradients. These results corroborate data from previous clinical trials and could have been caused by V/Q mismatch in the collapsed lower areas of the NOVENT group [38, 42, 43]. Mitigating atelectasis through the continuation of ventilation minimized the downstream wash out and impairment of surfactant production, ultimately alleviating the continued a-/dystelectasis, shunt and poor oxygenation [44]. Similar conclusions were drawn in a meta-analysis from Chi et al. who demonstrated that improvements in PF ratio and A-a gradient were attributed to the ventilation-mediated mitigation of atelectasis and V/Q mismatch [16].

The postoperative ARDS observed in the NOVENT group could have been exacerbated by VILI. The increased regional atelectasis and lower lobe collapse in the NOVENT group appears to have led to the uneven distribution of ventilation through the upper lobes during the postoperative monitoring period. Recruitment maneuvers performed at the end of surgery could have also contributed, as bag ventilation performed in the NOVENT group was more likely to cause overdistension due to the difficulty in measuring the exact volumes applied [45]. This is reflected in our findings of higher driving pressures in this group, signifying an increasing susceptibility to VILI and over inflation [44]. Of note, the chest wall was maintained open during the entire postoperative monitoring period, implying that all the pressure created by the ventilator was completely applied to isolated lungs. Hence, the values presented in this paper likely overestimate the extent of the driving pressure seen in typical patients, as the closure of the chest wall in these patients would prevent lung overinflation and excessive strain [46]. Furthermore, our histological lung analysis revealed the presence of hypertrophy in the intralobular septum and thickening of the space between alveolar lining, which can be due to increased strain and respiratory effort. Our results corroborate multiple studies that previously suggested high driving pressures can worsen patient outcome, through either alveolar overdistension, or the repetitive recruitment and re-collapse of unstable tissue in areas impacted by surfactant dysfunction [47–49]. Further, the risk of lung overinflation in cardiac patients is significantly more likely due to abnormal chest wall compliance following sternotomy [50]. To the best of our knowledge, this is the first report appraising driving pressure in this context. Furthermore, our results demonstrate an increase in neutrophil infiltration caused by lung damage associated with alveolar collapse in the lower lobes, as well as overdistension due to VILI in the upper regions. Several previous animal studies confirm this, suggesting that atelectasis-mediated epithelial damage and alveolar overdistension can separately recruit monocytes and neutrophils, and increase cytokine transcription [19, 51–54]. Conversely, ventilation during CPB appeared to lower driving pressures and immune cell infiltration, most likely through mitigation of atelectasis and reduction in VILI. IL-8 concentrations were expected to increase in the NOVENT group, however no differences were seen [55, 56]. Our findings could potentially be explained by the distinctive response of IL-8, a stress-responsive proinflammatory chemokine. Its activation facilitates the influx of neutrophils and subsequent inflammation. Alternatively, our negative results may have been related to the use of corticosteroids post-transplant or lung biopsies, which could potentially underestimate lobar inflammation. In future investigations, it would be prudent to conduct comprehensive evaluations encompassing both pro-inflammatory and anti-inflammatory chemokines. Additionally, employing bronchoalveolar lavage techniques could offer a more thorough characterization of ventilator-induced inflammation [57].

Our study has several noteworthy strengths. Firstly, we employed a clinically relevant sheep model of orthotopic heart transplantation performed 24 h after donor brainstem death. This model has proven to be highly translatable, as evidenced by the first Australian and New Zealand trial, which demonstrated that hypothermic oxygenated perfusion (HOPE) safely and effectively extends acceptable donor heart preservation times in humans [58]. Secondly, we utilized histological appraisal of lung injury rather than relying on surrogate endpoints of lung function. Lastly, we conducted a comprehensive evaluation of potential alternative causes of lung injury, including cardiac function assessed through echocardiography, inflammatory markers, and injurious ventilatory settings. Major limitations of the study included the relatively low number of samples. Secondly, this ventilation study was performed within the limitations of the HTx model and involved different heart preservation strategies and types of death in the donor sheep. These factors all introduced a degree of variability into the condition of the transplanted heart that could not be accounted for and may have influenced postoperative lung function, independent of perioperative ventilation. We introduced a randomized scheme to counter any potential bias, and the distribution of preservation methods between intervention groups was similar (Table E1). However, it is possible that the condition of the transplanted heart, and the influence of postoperative vasoactive support, remained a confounding factor. It must be noted that the sheep were immunosuppressed due to the use of corticosteroids post heart transplantation. This could have limited the accuracy of our IL-8 assays. Further, due to the nature of the animal model, no conclusions could be made regarding important clinical factors, such as time to extubation and length of ICU and hospital stays. While the study provided strong evidence for the use of ventilation during CPB, the restricted postoperative monitoring period limited our ability to determine whether initial ventilation-mediated improvements in respiratory function can lead to long-term benefits in patient outcome. These clinical outcomes have been investigated in the literature, with the PROVECS trial exploring protective ventilation during CPB, but no differences in postoperative complications or other endpoints have been observed [23]. The MECANO trial showed similar results, however the authors did note that ventilation during CPB significantly decreased postoperative complications in isolated patients undergoing CABG [24]. Nguyen and colleagues emphasized that these post hoc subgroup analyses carry less weight as compared to primary analysis, but are of clinical relevance and warrant further investigation [24]*.* As Schultz and colleagues note, both trials may have been underpowered to show benefit from the maintenance of ventilation, and further investigation is needed [59]. Thirdly, pulmonary perfusion data were not recorded during the study. Although cardiac output did not show a significant difference between groups, pulmonary artery pressure and resistance should be investigated in future studies to understand heart–lung interactions during mechanical ventilation. These parameters could have provided further insight into the extent of lung injury between the groups. Fourthly, additional markers of lung permeability, such as the wet-to-dry lung weight ratio and the lung-to-body weight ratio, were not reported. Since this was a concomitant study, the primary focus was on evaluating donor heart function in the HTx model, and lung assessment was a secondary objective. Lastly, a single senior veterinary pathologist performed the histology assessment of lung injury. While this could increase the risk of bias due to the lack of cross-verification and potential subjectivity, the pathologist was blinded to treatment allocation.

In conclusion, in our ovine HTx model, the continuation of ventilation during CPB significantly improved short-term postoperative oxygenation parameters including pulmonary shunt and A-a gradient, as well as reducing histological damage, inflammatory cell infiltration and driving pressures. These data provide perceptive new pre-clinical insights on the pathophysiology of lung derecruitment during cardiac transplantation and the value of an approach to avoid postoperative ARDS following cardiac surgery.

## Supplementary Information

Supplementary Material 1

## Data Availability

Raw data will be made available upon reasonable request.

## Abbreviations

ARDS: Acute respiratory distress syndrome
ARDS: Acute respiratory distress syndrome
BD: Brain stem death
BSL: Baseline recordings
CABG: Coronary artery bypass graft
CPB: Cardiopulmonary bypass
EDA: End-diastolic area
ELISA: Enzyme linked immunosorbent assay
EndoGCS: Endo-myocardial global circumferential strain
ESA: End-systolic area
ETCO: End tidal carbon dioxide
FAC: Fractional area change
FiO_2_: Fraction of inspired O_2_
GRS: Global radial strain
H&E: Hematoxylin and eosin
HOPE: Hypothermic oxygenated perfusion
HTx: Heart transplant
IHC: Immunohistochemistry
IL-8: Interleukin 8
IR: Ischemia–reperfusion
LLL: Left lower lobe
LTV: Low tidal volume
LUL: Left upper lobe
NHMRC: National Health and Medical Research Council
NOVENT: No ventilation
OPENVENT: Open-lung ventilation group
PAO_2_: Alveolar partial pressure of O_2_
PaO_2_: Arterial partial pressure of O_2_
PEEP: Positive end-expiratory pressure
PVENT: Protective ventilation
QQ: Quantile–quantile
RCTs: Randomized controlled trials
RLL: Right lower lobe
RML: Right middle lobe
RMs: Recruitment maneuvers
RR: Respiratory rate
RUL: Right upper lobe
SCS: Static cold storage
SIRS: Systemic inflammatory response syndrome
SPO_2_: Oxygen saturation
V/Q: Tidal volume
V_T_: Ventilation/perfusion ratio
VILI: Ventilator induced lung injury
