# Supplementary material for: Open-lung ventilation versus no ventilation during cardiopulmonary bypass in an innovative animal model of heart transplantation
Source: Intensive Care Med Exp. 2024 Nov 27;12:109. doi: 10.1186/s40635-024-00669-w (PMC11602927; doi:10.1186/s40635-024-00669-w)
Supplement: Supplementary file 1 — Supplementary Material 1 [file 40635_2024_669_MOESM1_ESM.docx]

## SUPPLEMENTARY MATERIAL

**Open-lung ventilation vs. no ventilation during cardiopulmonary bypass in an innovative animal model of heart transplantation**

**Authors:**

Varun Karnik Bsc (Hons)^1,2,3#^, Sebastiano Maria Colombo MD^1,3,4#^, Leah Rickards MBBS^5^, Silver Heinsar MD^1,3,8^, Louise E. See Hoe PhD^1,3,6^, Karin Wildi MD PhD^1,3,7^, Margaret R. Passmore BScHons^1,3^, Mahe Bouquet MbiomedSc^1,3^, Kei Sato MD^1,3^, Carmen Ainola RN Mphil^1,3^, Nicole Bartnikowski PhD^1,3,9^, Emily S. Wilson^1,3^, Kieran Hyslop PGDipSci^1,3^, Kris Skeggs MBBS^1,10^, Nchafatso G. Obonyo MB.ChB, PhD^1,3,11,12^, Charles McDonald PhD^1,14^, Samantha Livingstone BVSc, PhD^1,3^, Gabriella Abbate RN^1,3^, Andrew Haymet MBBS^1,3^, Jae-Seung Jung MD^1,3,15^, Noriko Sato RN^1,3^, Lynnette James RN^1,10^, Benjamin Lloyd MBBS, FANZCA^1,10^, Nicole White PhD^1,16^, Chiara Palmieri, DVM PhD DiplECVP^17^, Mark Buckland MD, FANZCA^18^, Jacky Y. Suen PhD^1,3,13,19^, David C. McGiffin MBBS, DmedHS^1,3,20,21^, John F. Fraser MB.ChB, PhD^1,3,22,23 *^, and Gianluigi Li Bassi MD, PhD^1,3,10,22-25*^

# VK and SMC equally contributed to this work

* JFF and GLB equally contributed to this work

## SUPPLEMENTARY METHODS

*Pulmonary Inflammation*

*Macrophage and Neutrophil Infiltration*

ImageJ was used for semi-quantitative analysis of neutrophil infiltration, with the neutrophil count expressed as the logarithm of the absolute count, over twenty fields of view. Macrophage infiltration was similar semi-quantified, facilitated by immunohistochemistry (IHC). Sections of lung tissue underwent dewaxing and rehydrating in a series of xylene, ethanol, and water baths. Samples were brought to near-boiling in a sodium citrate buffer for antigen retrieval. Following this, peroxidase and normal serum-blocking solutions were applied to the slides to mitigate background staining from endogenous and non-specific factors. Slides were coated with primary macrophage antibody Cat#000-06455 [MAC 387] (Genetex, CA, USA) and incubated overnight at 4°C. After washing the sections, the Vector universal ABC secondary antibody kit Cat#PK-6200 (Vector Laboratories, CA, USA) was used to intensify the detection signal. A reaction involving 3,3’-diaminobenzidine tetrahydrochloride Cat#D5905 (Sigma-Aldrich, St Louis, USA) produced a brown color at the site of the target protein. Negative control was performed by omitting the primary antibody, indicating the extent of background staining. Following haematoxylin counterstaining, the sections were dehydrated with a series of ethanol and xylene baths and mounted in DPX media Cat#06522 (Sigma- Aldrich, St Louis, USA). Macrophage quantification was performed with ImageJ, with average cell counts over twenty fields of view expressed as percentage of total cells.

*Inflammatory Cytokine Quantification*

*Homogenization and BCA*

Frozen samples were dethawed, and 100mg was weighed. An extraction buffer, consisting of T-PER (Thermofisher Scientific Cat #78510) and a protease/phosphate inhibitor cocktail (Abcam Cat# ab201119) was added (1:9 ratio) to the samples. After homogenization by the

Polytron (PT 2100 benchtop homogenizer, Kinematica, Switzerland), samples were centrifuged (5 min at 10 000 RPM at 4 °C), and the supernatant was isolated. A Pierce BCA Protein Assay kit (Cat# 23225, ThermoFisher Scientific, MA, USA) was then used for colorimetric detection and quantification of total protein in each sample. Protein concentrations were determined and reported with reference to a standard, in this case, bovine serum albumin (BSA). A series of dilutions of known BSA concentrations were prepared and assayed alongside the unknown samples. The optical density of each well was determined using a microplate reader (FLUOstar Omega, BMG Labtech, VIC, Australia) set to 562 nm. Duplicate readings of each standard and samples were averaged and subtracted the zero standard optical density. A standard curve was created and a four-parameter logistic (4-PL) curve fit used to determine the total protein concentration in each sample (GraphPad Prism 8, CA, USA). According to these results, samples were diluted to a final concentration of 3000 μg/mL total protein.

*Enzyme Linked Immunosorbent Assays (ELISA)*

In-house ELISA were performed to detect inflammatory cytokine IL-8 (1). Firstly, clear plates (Nunc Maxisorp; ThermoFisher Scientific, MA, USA) were coated with capture antibody. Following overnight incubation at 4°C, the plates were washed using an ELx50 plate washer (BioTek, VT, USA). The blocking buffer, samples, and antibodies were added sequentially, with washes performed between each step. Visualization was facilitated by 3, 3′, 5, 5′-tetramethylbenzidine (TMB) (Sigma-Aldrich), with 1M H2SO4 used to stop the reaction and a BMG Labtech microplate reader (VIC, Australia) used to determine the absorbance at both 450nm and 670nm. Graphpad Prism 8 was used to determine concentrations in samples and the standards, utilising a standard curve and a four-parameter logistic (4-PL) curve fit. The positive control was performed by utilising a recombinant protein specific to each cytokine, while a blank well was used as the negative control.

*Functional Measurements of Gas Exchange and Respiratory Mechanics*

Ventilatory parameters were recorded via the Te Hamilton-G5 ventilator at 100Hz. Firstly, arterial partial pressures of O_2_ (PaO_2_) and CO_2_ (PaCO_2_) were determined by blood gas analysis. The inspired oxygen fraction (FiO_2_) was indicated by the ventilator, while alveolar O_2_ partial pressure (PAO_2_) was estimated using the alveolar gas equation (Equation 1). P_atmos_ and P_H2O_ were assumed to be 760mmHg and 47mmHg respectively and were used to estimate the percentage of FiO_2_ that reached the alveoli after humidification in the airways. These values allowed the determination of common respiratory parameters, the PAO_2_-PaO_2_ (A-a) gradient and the PaO_2_/FiO_2_ ratio.

$$P_{A}O_{2}=\left( F_{I}O_{2}\times\left( P_{Atmos}- P_{H_{2}O} \right) \right)- \frac{P_{a}CO_{2}}{R}$$

*Equation 1: Calculating Alveolar Gas (*$P_{A}O_{2}$ *= Alveolar O_2_ partial pressure,* $F_{I}O_{2}$*= inspired O_2_ partial pressure,* $P_{Atmos}= atmospheric pressure, P_{H_{2}O}=vapour pressure$

$in alveolus,$ $P_{a}CO_{2}$ *= arterial CO_2_ partial pressure,* $R$ *= respiratory quotient (0.8))*

Pulmonary shunt was calculated by first converting O_2_ partial pressure values from arterial and mixed venous blood gas readings into oxygen content (Equation 2)

$$C_{x}O_{2}=Dissolved Oxygen+Oxygen delivered by Hb$$

$$C_{x}O_{2}=(0.003 \times P_{x}O_{2})+(1.34 \times\left[ Hb \right]\times SHbO_{2})$$

*Equation 2: Calculating Oxygen Content (*$x$ *= arterial/mixed venous,*$\left[ Hb \right]$ *= concentration of haemoglobin,* $SHbO_{2}$ *= percentage of haemoglobin that is bound with oxygen)*

Next, to calculate the ideal end capillary O_2_ content, capillary partial pressure values of O_2_ were assumed to be identical to alveolar O_2_ partial pressure, and hence were calculated by the alveolar gas equation (Equation 1). These values were then converted into oxygen content (Equation 2). Values of oxygen content were used in the shunt fraction equation, to calculate the pulmonary shunt (Equation 3).

$$\frac{Q_{s}}{Q_{t}}=\frac{C_{c}O_{2}-C_{a}O_{2}}{C_{c}O_{2}-C_{v}O_{2}}$$

*Equation 3: Calculating Shunt Fraction (*$\frac{Q_{s}}{Q_{t}}$ *= Shunt fraction,* $C_{c}O_{2}$ *= end capillary O_2_ content,* $C_{a}O_{2}$ *= arterial O_2_ content,* $C_{v}O_{2}$ *= venous O_2_ content)*

The ventilator indicated the CO_2_ partial pressure in the exhaled air (ETCO_2_), which was used in conjunction with the PaCO_2_ to calculate the physiological dead space, via the Bohr Equation (Equation 4).

$$V_{d}=V_{T}\times\frac{P_{a}{CO}_{2}-ETCO_{2}}{P_{a}{CO}_{2}}$$

*Equation 4: Calculating Physiological Dead Space (*$V_{D}$ *= Dead Space Volume,* $V_{T}$ *= Tidal Volume,* $P_{a}CO_{2}$*= arterial CO_2_ partial pressure,* $ET{CO}_{2}$*= expired CO_2_ partial pressure)*

Driving pressure was calculated by calculating the difference between plateau pressure and positive end expiratory pressure (PEEP), both of which were indicated by the ventilator.

$$Driving Pressure = Plateau Pressure-{PEEP}_{Total}$$

*Equation 5: Calculating Driving Pressure (PEEP = Positive End Expiratory Pressure)*

The static compliance of the lung tissue was determined by the compliance equation, using the driving pressure calculated above (Equation 6).

$$Lung Compliance= \frac{V_{T}}{Driving Pressure}$$

*Equation 6: Calculating Lung Compliance (*$V_{T}$ *= Tidal Volume)*

Oxygen delivery and consumption were calculated using the equations below.

$$Oxygen Delivery= CO \times C_{a}O_{2}$$

*Equation 7: Calculating Oxygen Delivery (*$CO$ *= Cardiac Output,* $C_{a}O_{2}$ *= arterial O_2_ content)*

$$Oxygen Consumption= CO \times(C_{a}O_{2} -C_{v}O_{2})$$

*Equation 8: Calculating Oxygen Consumption (*$CO$ *= Cardiac Output,* $C_{a}O_{2}$ *= arterial O_2_ content,* $C_{v}O_{2}$ *= venous O_2_ content)*

CO_2_ arterial – venous gradient and Ventilatory Ratio were calculated using the equations below. The Ventilatory Ratio Calculation was adapted from Sinha et al (26).

$$CO_{2} arterial-venous gradient= P_{a}CO_{2} -P_{v}CO_{2}$$

*Equation 9: Calculating CO_2_ arterial-venous gradient* ${(P}_{a}CO_{2}$ *= Arterial CO_2_ partial pressure,* $P_{v}CO_{2}$ *= Venous CO_2_ partial pressure)*

$$Ventilatory Ratio=\frac{(V_{e}\times P_{a}CO_{2})}{Predicted V_{e} \times Predicted P_{a}CO_{2}}$$

*Equation 10: Ventilatory Ratio* ${(P}_{a}CO_{2}$ *= Arterial CO_2_ partial pressure,* $V_{e}$ *= Minute Volume,* $Predicted V_{e}$*= Bodyweight* $\times$ *100,* $Predicted P_{a}CO_{2}$*= 37.5* $\times$ $Predicted V_{e}$

*Epicardial Echocardiography*

Three-beat ECG-gated loops were acquired using the conventional parasternal short axis (PSAX) view. Anatomic constraints from the sternotomy resulted in conventional apical views not being able to be obtained. TomTec-Arena (TomTec imaging Systems GMBH, Unterschleim, Germany) was used on separate workstation to analyse the images offline. Automated Functional Imaging (AFI) was then applied to echo-loops in the PSAX view. Speckle tracking was visually assessed for tracking accuracy, and if necessary, the end-diastolic timing marker was manually adjusted. Data was collected for end-diastolic area (EDA), end-systolic area (ESA), fractional area change (FAC), endo-myocardial global circumferential strain (EndoGCS) and global radial strain (GRS). The calculation for FAC is given below, and the GRS was defined as strain derived from the whole thickness of the myocardium in the radial direction.

$$FAC =\frac{EDA-ESA}{EDA}$$

*Equation 11: Calculating fractional area change (*$FAC$ *= fractional area change,* $EDA$*= end-diastolic area,* $ESA$*= end-systolic area)*

## PRIMARY OUTCOME POST-HOC POWER ANALYSIS

For the mixed model simulation based on the current sample size, the post-hoc power was 37.00% (95% CI 27.56, 47.24). The calculation compares a model with and without ‘group’ included as a fixed effect.

Increasing the number of animals to 20 per group resulted in an increase in power of 80.30% (95% CI 77.70, 82.72)

Increasing the number of animals to 25 per group resulted in an increase in power of 86.00% (95% CI 77.63, 92.13)

Increasing the number of animals to 30 per group resulted in an increase in power of 92.60% (95% CI 90.80, 94.15)

## SUPPLEMENTARY FIGURE LEGENDS

**Figure E1**: (A) Oxygen Consumption (L/ min), (B) Oxygen Delivery (mL/min), (C) Arterial Lactate (mmol/L) and (D) Arterial Base Excess (mmol/L) during 6-hour post-transplantation monitoring period in recipient sheep treated with either no ventilation (NOVENT ■) or open-lung ventilation (OPENVENT ●) during cardiopulmonary bypass. Data shown as Mean ± SEM from pre-operative baseline (BSL) through to 6 hours postoperative (T6), with n = 9 for both groups. F (a, b) = c, *a* represents the between group variance, *b* the within group variance, the F value (*c*) is the ratio of the variation between sample means/ variation within samples. Top F and p values refer to effect of ventilation strategy on outcome, and bottom F and p values refer to the combined effects of ventilation strategies and time (interaction term).

**Figure E2:** (A) FiO2 (%), (B) Ventilatory Ratio and (C) Respiratory Rate (breaths/min) during 6-hour post-transplantation monitoring period in recipient sheep treated with either no ventilation (NOVENT ■) or open-lung ventilation (OPENVENT ●) during cardiopulmonary bypass. Data shown as Mean ± SEM from pre-operative baseline (BSL) through to 6 hours postoperative (T6), with n = 9 for both groups. F (a, b) = c, *a* represents the between group variance, *b* the within group variance, the F value (*c*) is the ratio of the variation between sample means/ variation within samples. Top F and p values refer to effect of ventilation strategy on outcome, and bottom F and p values refer to the combined effects of ventilation strategies and time (interaction term).

**FIGURE E1**


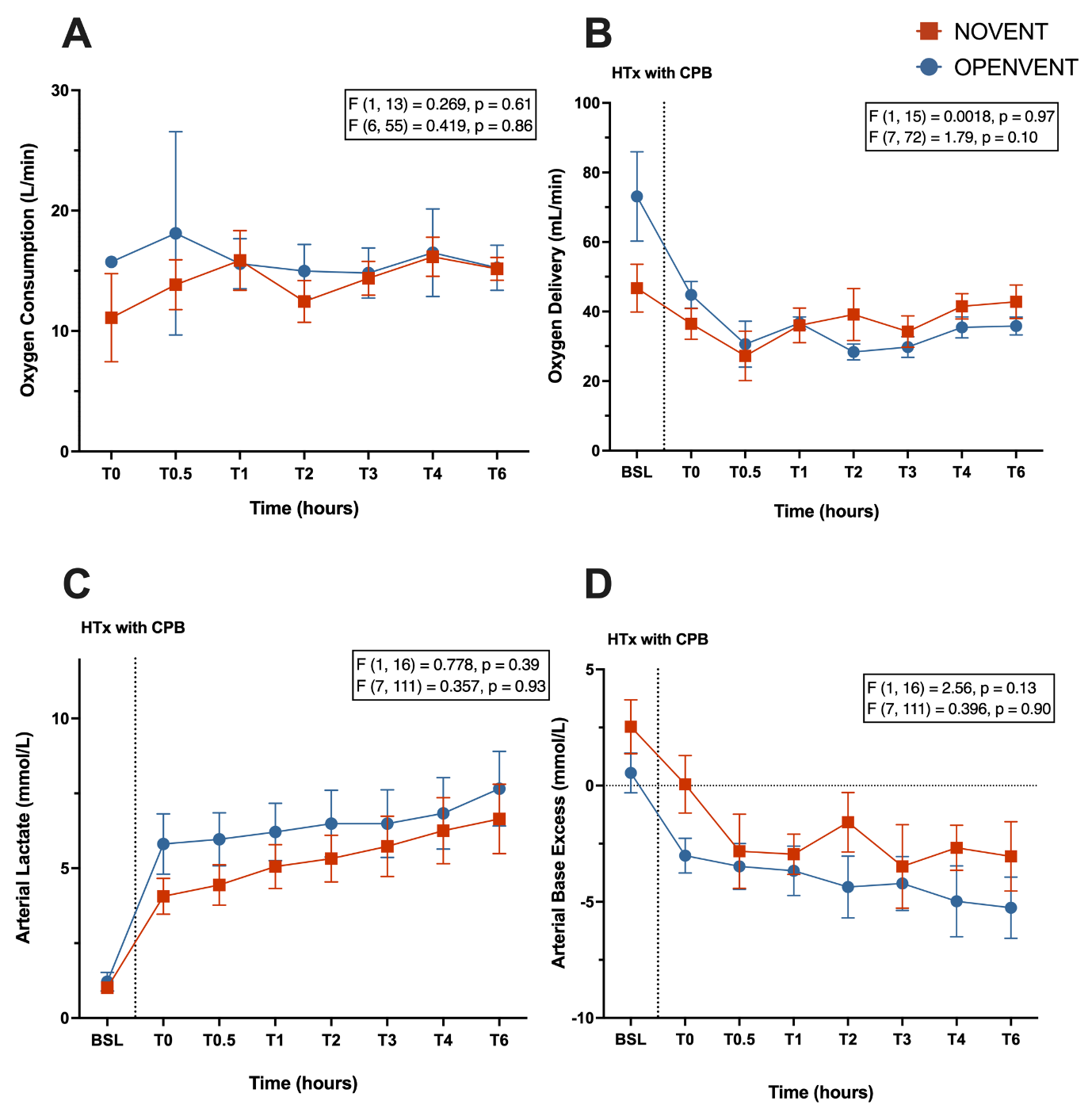


**FIGURE E2**


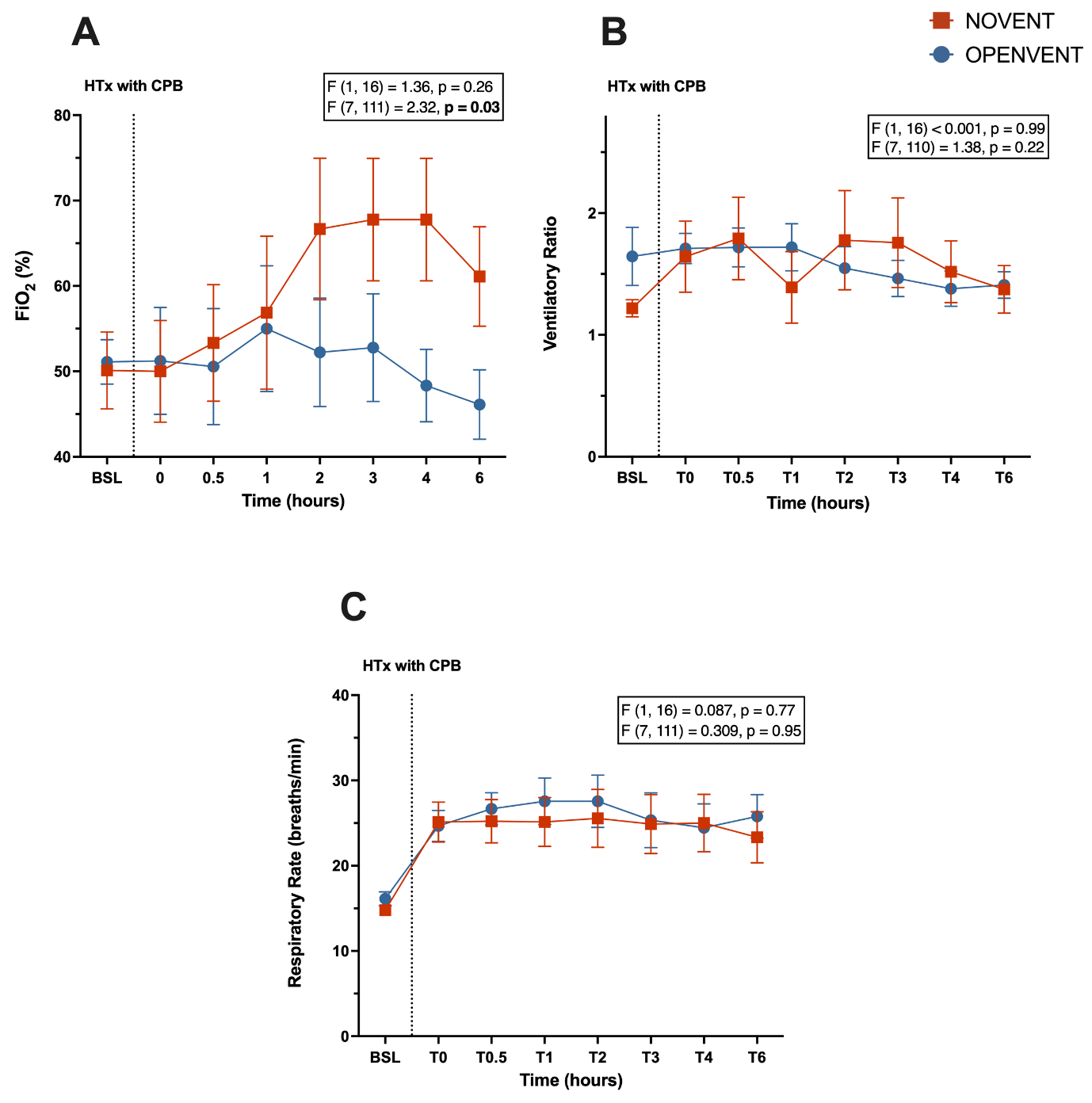


## SUPPLEMENTARY TABLES

**Table E1 Caption:** Distribution of preservation methods to intervention groups. HOPE, hypothermic oxygenated perfusion; SCS, static cold storage

| Intervention Group | NOVENT | OPENVENT |
| --- | --- | --- |
| Sheep ID and Preservation method | 1878 (HOPE) | 1887 (SCS) |
|  | 1906 (8hr HOPE) | 1897 (2hr HOPE) |
|  | 1911 (8hr HOPE) | 1914 (2hr HOPE) |
|  | 1907 (8hr HOPE) | 1913 (2hr HOPE) |
|  | 1922 (2hr HOPE) | 1934 (8hr HOPE) |
|  | 1929 (2hr HOPE) | 1938 (8hr HOPE) |
|  | 1963 (8hr HOPE) | 1965 (8hr HOPE) |
|  | 1962 (8hr HOPE) | 1955 (8hr HOPE) |
|  | 1961 (SCS) | 1959 (SCS) |

**Table E2 Caption:** Lung Histological Damage Scoring System adapted from Kulkarni et al (2)

|  | Score per field | | |
| --- | --- | --- | --- |
| Parameter | 0 | 1 | 2 |
| Proteinaceous debris in the airspaces (e.g. fibrin and/or proteinaceous material) | none | <50% alveoli | >50% alveoli |
| Evidence of alveolar epithelial injury (cell death, epithelial denudation, ATII proliferation) | none | <50% alveoli | >50% alveoli |
| Neutrophils in the alveolar space | none | <10 | >10 |
| Thickening of the alveolar septa (interstitial oedema or congestion) | <2x | 2x-4x | >4x |
| Diffuse alveolar damage pattern (hyaline membranes) | none | 1 | >1 |
| Intraalveolar haemorrhages | none | <50% alveoli | >50% alveoli |
| Evidence of capillary and/or endothelial cell death (vascular damage) | none | 1 vessel affected | >1 vessel affected |
| Neutrophils in the interstitial space (within the interstitium and circulating or adhering to the alveolar capillaries) | none | <10 | >10 |
| Thrombi within capillaries/small blood vessels (40x) | none | 1 | >1 |
| Atelectasis | none | <50% of the section | >50% of the section |
| Hypertrophy of the septal muscle | none | <50% alveoli | >50% alveoli |

**REFERENCES**

1. Bouquet M, Passmore MR, See Hoe LE, Tung J-P, Simonova G, Boon A-C, et al. Development and validation of ELISAs for the quantitation of interleukin (IL)-1β, IL-6, IL-8 and IL-10 in ovine plasma. Journal of Immunological Methods. 2020:112835.

2. Kulkarni HS, Lee JS, Bastarache JA, Kuebler WM, Downey GP, Albaiceta GM, et al. Update on the Features and Measurements of Experimental Acute Lung Injury in Animals: An Official American Thoracic Society Workshop Report. Am J Respir Cell Mol Biol. 2022;66(2):e1-e14.
